# Druggists’ Association

**Published:** 1880-07

**Authors:** 


### DRUGGISTS' ASSOCIATION.

A MEETING of the principal druggists in this city was held at the Tifft House on the 4th of June, and resolutions were adopted favoring a permanent organization. One committee was appointed to draft a constitution, by-laws and code of ethics, and another to secure a place for future meetings. It was furthermore resolved, "That we warmly endorse the action of our representatives at Syracuse in inviting the State Association to Buffalo in 1880, and assure them of our most hearty coöperation in creditably entertaining the visitors."

Such an organization must ultimately prove an advantage and a credit to the druggists of the city, and we congratulate them in this evidence of their zeal.

THE nineteenth volume of this JOURNAL is completed with the present number, and while the editors congratulate themselves upon a certain amount of work accomplished, they also feel grateful to those collaborators who have contributed to its success. There is much satisfaction to be derived from the fact that the circulation of the JOURNAL has been very largely increased during the past year, an indication at least that the efforts are not unappreciated, whose tendency is to raise the standard of perfection ever higher and higher. The volume closes with an unusually complete index, which must prove of great assistance to constant readers, and a decided convenience to those who would wish even occasionally to consult its pages.

---

WE take pleasure in calling the attention of our friends to the advertisement in this issue of *Johnston's Fluid Beef.* It has received the warm commendation of many of the highest authorities in Great Britain and Ireland, as containing all the nutritive constituents of meat, and as supplying in the most easily digested form all the material necessary for renewing the tissues wasted by disease. It is now used largely by the United States government, and has been taken up by the Massachusetts General and the City Hospital, Boston, and many of the best hospitals in New York and Philadelphia.

